# The MOBI-Kids Study Protocol: Challenges in Assessing Childhood and Adolescent Exposure to Electromagnetic Fields from Wireless Telecommunication Technologies and Possible Association with Brain Tumor Risk

**DOI:** 10.3389/fpubh.2014.00124

**Published:** 2014-09-23

**Authors:** Siegal Sadetzki, Chelsea Eastman Langer, Revital Bruchim, Michael Kundi, Franco Merletti, Roel Vermeulen, Hans Kromhout, Ae-Kyoung Lee, Myron Maslanyj, Malcolm R. Sim, Masao Taki, Joe Wiart, Bruce Armstrong, Elizabeth Milne, Geza Benke, Rosa Schattner, Hans-Peter Hutter, Adelheid Woehrer, Daniel Krewski, Charmaine Mohipp, Franco Momoli, Paul Ritvo, John Spinelli, Brigitte Lacour, Dominique Delmas, Thomas Remen, Katja Radon, Tobias Weinmann, Swaantje Klostermann, Sabine Heinrich, Eleni Petridou, Evdoxia Bouka, Paraskevi Panagopoulou, Rajesh Dikshit, Rajini Nagrani, Hadas Even-Nir, Angela Chetrit, Milena Maule, Enrica Migliore, Graziella Filippini, Lucia Miligi, Stefano Mattioli, Naohito Yamaguchi, Noriko Kojimahara, Mina Ha, Kyung-Hwa Choi, Andrea ’t Mannetje, Amanda Eng, Alistair Woodward, Gema Carretero, Juan Alguacil, Nuria Aragones, Maria Morales Suare-Varela, Geertje Goedhart, A. Antoinette Y. N. Schouten-van Meeteren, A. Ardine M. J. Reedijk, Elisabeth Cardis

**Affiliations:** ^1^Cancer and Radiation Epidemiology Unit, Gertner Institute, Chaim Sheba Medical Center, Ramat Gan, Israel; ^2^Sackler Faculty of Medicine, Tel Aviv University, Tel Aviv, Israel; ^3^Centre for Research in Environmental Epidemiology (CREAL), Barcelona, Spain; ^4^Universitat Pompeu Fabra (UPF), Barcelona, Spain; ^5^Ciber Epidemiología y Salud Pública (CIBERESP), Barcelona, Spain; ^6^Center for Public Health, Institute of Environmental Health, Medical University Vienna, Vienna, Austria; ^7^Unit of Cancer Epidemiology, Department of Medical Sciences, University of Turin, Turin, Italy; ^8^Division Environmental Epidemiology, Institute for Risk Assessment Sciences, Utrecht University, Utrecht, Netherlands; ^9^Radio Technology Research Department, Electronics and Telecommunication Research Institute (ETRI), Daejeon, South Korea; ^10^Centre for Radiation, Chemical and Environmental Hazards, Public Health England, Chilton, UK; ^11^Department of Epidemiology and Preventive Medicine, Monash University, Melbourne, VIC, Australia; ^12^Department of Electrical Engineering, Tokyo Metropolitan University, Tokyo, Japan; ^13^Whist Laboratory, Paris, France; ^14^The University of Sydney, Sydney, NSW, Australia; ^15^Telecom Institute for Child Health Research Western Australia, Perth, WA, Australia; ^16^Clinical Institute of Neurology, Medical University Vienna, Vienna, Austria; ^17^McLaughlin Centre for Population Health Risk Assessment, Institute of Population Health, Ottawa, ON, Canada; ^18^Department of Epidemiology and Community Medicine, Faculty of Medicine, University of Ottawa, Ottawa, ON, Canada; ^19^Children’s Hospital of Eastern Ontario, Ottawa, ON, Canada; ^20^University of Ottawa, Ottawa, ON, Canada; ^21^Ottawa Hospital Research Institute, Ottawa, ON, Canada; ^22^Children’s Hospital of Eastern Ontario Research Institute, Ottawa, ON, Canada; ^23^Research, Prevention and Cancer Control, Cancer Care Ontario, Ontario, ON, Canada; ^24^Cancer Control Research, British Columbia Cancer Agency, Vancouver, BC, Canada; ^25^French National Registry of Childhood Solid Tumors, CHU, Nancy, France; ^26^UMRS 1018, CESP, INSERM, Villejuif, France; ^27^Institute and Outpatient Clinic for Occupational, Social and Environmental Medicine, University Hospital of Munich (LMU), Munich, Germany; ^28^Department of Hygiene, Epidemiology and Medical Statistics, Medical School, National and Kapodistrian University of Athens, Athens, Greece; ^29^Centre for Cancer Epidemiology, Tata Memorial Centre, Mumbai, India; ^30^Unit of Cancer Epidemiology, Citta’ della Salute e della Scienza, University of Turin, Turin, Italy; ^31^Neuroepidemiology Research Unit, Instituto Nazionale Neurologico C. Besta, Milan, Italy; ^32^Unit of Occupational and Environmental Epidemiology, Institute for the Study and Prevention of Cancer, Florence, Italy; ^33^Department of Medical and Surgical Sciences, University of Bologna, Bologna, Italy; ^34^Department of Public Health, Tokyo Women’s Medical University, Tokyo, Japan; ^35^Department of Preventive Medicine, Dankook University College of Medicine, Cheonan, South Korea; ^36^Department of Public Health, Graduate School of Dankook University, Cheonan, South Korea; ^37^Centre for Public Health Research, Massey University, Wellington, New Zealand; ^38^School of Population Health, University of Auckland, Auckland, New Zealand; ^39^Centro de Investigación en Salud y Medio Ambiente (CYSMA), Universidad de Huelva, Huelva, Spain; ^40^Ciber Epidemiología y Salud Pública (CIBERESP), Huelva, Spain; ^41^Cancer and Environmental Epidemiology Area, National Center for Epidemiology, Carlos III Institute of Health, Madrid, Spain; ^42^Ciber Epidemiología y Salud Pública (CIBERESP), Madrid, Spain; ^43^Área de Medicina Preventiva y Salud Pública, Universitat de Valencia, Valencia, Spain; ^44^Department of Paediatric Oncology, Academic Medical Center, Emma Children’s Hospital, University of Amsterdam, Amsterdam, Netherlands; ^45^Dutch Childhood Oncology Group (DCOG), Den Haag, Netherlands

**Keywords:** children, adolescents, brain tumors, ELF–EMF, mobile phones, RF-EMF

## Abstract

The rapid increase in mobile phone use in young people has generated concern about possible health effects of exposure to radiofrequency (RF) and extremely low frequency (ELF) electromagnetic fields (EMF). MOBI-Kids, a multinational case–control study, investigates the potential effects of childhood and adolescent exposure to EMF from mobile communications technologies on brain tumor risk in 14 countries. The study, which aims to include approximately 1,000 brain tumor cases aged 10–24 years and two individually matched controls for each case, follows a common protocol and builds upon the methodological experience of the INTERPHONE study. The design and conduct of a study on EMF exposure and brain tumor risk in young people in a large number of countries is complex and poses methodological challenges. This manuscript discusses the design of MOBI-Kids and describes the challenges and approaches chosen to address them, including: (1) the choice of controls operated for suspected appendicitis, to reduce potential selection bias related to low response rates among population controls; (2) investigating a young study population spanning a relatively wide age range; (3) conducting a large, multinational epidemiological study, while adhering to increasingly stricter ethics requirements; (4) investigating a rare and potentially fatal disease; and (5) assessing exposure to EMF from communication technologies. Our experience in thus far developing and implementing the study protocol indicates that MOBI-Kids is feasible and will generate results that will contribute to the understanding of potential brain tumor risks associated with use of mobile phones and other wireless communications technologies among young people.

## Introduction

A number of national and international organizations have reviewed potential health effects of radiofrequency (RF) field and identified research gaps (1–7). In 2011, the International Agency for Research on Cancer (IARC) of the World Health Organization (WHO) classified RF fields as “possibly carcinogenic to humans – 2B” (7), a classification confirmed subsequently by the EU funded European Health Risk Assessment Network on Electromagnetic Fields Exposure (EFHRAN) (5).

The rapid worldwide increase in mobile phone use in adolescents and, more recently, children has generated additional interest in the possible health effects of exposure to RF [EU funding calls ENV.2008.1.2.1.1. “Health impacts of exposure to radiofrequency fields in childhood and adolescence” and ENV.2013.6.4-2 “Closing gaps of knowledge and reducing exposure to electromagnetic fields (EMF)”]. Concern particularly relating to children and adolescents originates from the likelihood that, if an increased risk exists, it could be greater for exposure at younger ages due to: increased sensitivity of the developing neurological system to effects of RF signals; higher estimated specific absorption rate (SAR) in children (due to a thinner skull and ears compared to adults) (8); and greater lifetime cumulative exposure compared to those who began mobile phone use in adulthood.

MOBI-Kids, a multinational case–control study, was therefore initiated to assess the potential effects of exposure to RF and of extremely low frequency (ELF) electromagnetic fields (EMF) from mobile phones on the development of central nervous system tumors among young people. This study builds upon the methodological experience of INTERPHONE, the 13-country collaborative effort investigating the possible association between mobile phone use and risk of gliomas, meningiomas, acoustic neurinomas, and parotid gland tumors among adults diagnosed during 2000–2004 (9–11). In designing MOBI-Kids, considerable effort was invested in improving the INTERPHONE design and adapting it to changing communication technologies and a younger age range. Quantitative exposure assessment is being improved by a group of researchers experienced in non-ionizing radiation, environmental, and occupational exposure assessment.

MOBI-Kids is the largest study to date investigating the potential association between mobile phone use and the risk of brain tumors among young people. To date, only one study, CEFALO (12), focused specifically on the possible association between mobile phone use and brain tumors among the young. No evidence of an increased brain tumor risk in association with years of use of mobile phones or cumulative call time was found among 352 cases diagnosed between 2004 and 2008. However, subjects in CEFALO were young (the median age at diagnosis was 13 years), and were not long-term or heavy users (the median period of use was 2.7 years). Large-scale ongoing studies of mobile phones are under way, notably the COSMOS study (13), but are restricted to adults.

This paper describes the study design of MOBI-Kids and the challenges encountered and solutions sought while developing the protocol with respect to: (1), choosing a representative control group while ensuring a high compliance rate under the chosen case–control design; (2) investigating a young study population spanning a relatively wide age range (10–24 years), with heterogeneous distributions of tumors and patterns of mobile phone use; (3) conducting a large, multinational epidemiological study while following increasingly strict ethics requirements; (4) investigating a rare and potentially fatal disease; and (5) assessing exposure to RF and ELF fields from changing communication technologies. Where useful, data collected in the study up until June 2014 are used to illustrate the study methods and associated challenges.

## Study Design

MOBI-Kids is a prospective case–control study conducted in 14 countries: Australia, Austria, Canada, France, Germany, Greece, India, Israel, Italy, Japan, Korea, New Zealand, Spain, and The Netherlands. As brain tumors in young people are rare, and because the effect of EMF from mobile phones, if any, is probably weak, MOBI-Kids was designed as a multinational collaboration spanning a target population of almost 40 million individuals (Table 1). The case–control design was chosen, as a cohort study with similar statistical power would be extremely expensive requiring a similarly sized population with many years of follow-up.

**Table 1:** **Description of selected characteristics of the study population and design by center as of December 2013**.

Study center	Study region	Diagnostic period		Number of participating hospitals		No. of ethics committees (e.g., IRBs) needed to obtain ethics for all participating hospitals	Direct access to data on eligible patients periodically or in the end of the study	Expected number of eligible cases	
		Start	End	Cases	Controls			Target population at risk	Expected eligible cases (per year)
Australia	Greater metropolitan areas of Melbourne and Sydney	June 2012	December 2014	10	26	25	No	1,600,000	32
Austria	Nationwide	June 2012	December 2014	4	7	10	Yes	1,500,000	30
Canada	Greater metropolitan areas of Ottawa, Toronto, and Vancouver	June 2012	December 2014	7	12	15	Yes	1,760,905	54
France	15 Districts in 7 areas: Lorraine, Ile-de-France, Rhône-Isère, Hérault, Bouches-du-Rhône, Alsace, Gironde	March 2011	December 2014	14	44	1	Yes	3,485,577	63
Germany	Nationwide	October 2010	June 2014	62	65	8	No	5,598,131	84
Greece	Nationwide	May 2010	December 2013	23	19	16^a^	No	1,690,000	16
India	Mumbai	May 2013	December 2014	2	2	2	Yes	4,358,085	28
Israel	Nationwide	August 2010	December 2014	5	10	10	Yes	1,800,000	27
Italy	Four regions: Piemonte, Lombardia, Toscana, and Emilia Romagna	January 2011	September 2014	33	39	45	Yes	2,937,400	46
Japan	Tokyo metropolitan area	June 2011	December 2014	18	13	23	No	1,700,000	34
Korea	Metropolitan areas of Seoul and Incheon and Gyeonggi-do province	January 2012	December 2014	5	10	8	No	4,713,814	52
New Zealand	Nationwide	June 2013	December 2014	–	–	–	Yes	925,720	17
Spain	Four autonomous communities: Andalucia, Catalonia, Madrid, and Valencia	January 2011	December 2014	58	69	69	Yes	4,134,986	43
The Netherlands	Nationwide	June 2011	December 2014	10	7	17	No	1,600,000	30
**Total**	**–**	**May 2010**	**December 2014**	**251**	**323**	**249**	**–**	**37,804,618**	**556**

## Study Population

The target study population consists of all males and females aged 10–24 years residing in the study region with a confirmed diagnosis of an eligible first primary brain tumor diagnosed during the study period. In some countries, the study region encompasses the entire country, while in others it is restricted to defined areas (usually the major metropolitan areas) (Table 1). The period of case ascertainment varies by country, first beginning in mid-2010 and continuing through 2014.

The age range was an important consideration in defining the study population. The most likely mechanism by which exposure to ELF and RF-EMF may increase the risk of cancer is through a tumor promotion or progression effect (14, 15). Therefore, in the case of exposure to ELF and RF-EMF, a relatively short latency period may be a reasonable assumption, particularly, for tumors in young people. However, we had to ensure that the prevalence of mobile phone use in the past would be sufficient for the study to have adequate statistical power. Given the historically low use of mobile phones in children below the age of 12 years and the comparatively high use in teenagers and young adults, a study of brain tumors in subjects aged 15–24 years would have the most power to evaluate tumor risk from mobile phone use in young adults. However, as mobile phones have become increasing popular among 8–10 year olds since 2005 (16), and because of the expanding number of other sources of RF signals in the home (e.g., Wi-Fi), it is also of interest to study tumors in children aged 10–14 years. There appears to be little benefit in including younger subjects in this study due to their limited use of mobile phones. The relatively wide age range of the study population (encompassing both children and adults) raises issues, however, such as the need to design a questionnaire that is clear to the entire age range and to properly separate questions to be answered by parents and by the subjects themselves. Further, covering the ages of 10–24 requires including both adult and pediatric services, complicating ethics board approvals, and study logistics.

## Case Definition

Eligible diagnoses include only tumors originating in parts of the brain likely to experience the highest exposure to RF-EMF from mobile phones, which mainly comprises tumors not located in the midline (Supplementary Material) (17). Both benign and malignant brain tumors (not only gliomas and meningiomas but also many other tumor types) are included in the study. This histological heterogeneity of tumors could dilute a carcinogenic effect, if it exists, for a specific type of brain tumor. However, considering the rarity of these tumors among children, a separate analysis for each tumor type (apart from glioma and by grade of malignancy) will likely be unfeasible. A case is excluded if s/he has insufficient knowledge of the study language(s) and/or a known genetic syndrome related to brain tumors (e.g., neurofibromatosis).

The original expected number of cases in the target age range was of the order of 2,000. With the implementation of the study, however, it became apparent that the number of eligible cases is, in fact, much lower, in large part due to an underestimation of the number of midline tumors in the study population and, to a lesser extent, the failure of busy medical staff to notify eligible patients in some centers. In most centers, it is difficult to know exactly how many cases are ineligible as doctors/hospital staff will generally not inform study staff of ineligible cases. However, centers with access to detailed, reliable registry information or hospital records have excluded from one-third to more than one-half of cases due to an ineligible (midline) diagnosis. Table 1 indicates the revised expected number of eligible cases per year; the revised expected total number of cases to be included in MOBI-Kids is around 1,000, based on each center’s length of time in the field and other factors such as number of participating hospitals and accessibility to eligible cases. Fortunately, the MOBI-Kids study still has sufficient statistical power despite the reduced number of cases (see Study Power below).

## Selection of Controls

Two hospital-based controls (who underwent an appendectomy for suspected diagnosis of appendicitis) are selected for each case, and matched on: sex; age (±1 year for cases younger than 17 years and ±2 years for cases 17 years and older); date of surgery/interview (±3 months); and geographic area of residence. In centers experiencing difficulties recruiting controls under the above criteria, the protocol was modified to allow more flexibility: date of surgery (±4 months); expanded area of residence (at the center’s discretion); and broader age range (an additional 6 months). In addition to the cases’ exclusion criteria, controls are excluded if the interviewer decides they are mentally unable to understand and answer the questions. Care is taken to select controls from the same population base as the cases. Since cases are identified from tertiary centers, many more hospitals must participate in the identification of controls to cover the catchment area from which cases may arise.

The rationale for appendicitis patients was the inherent difficulty in case–control studies to recruit representative controls, which is essential to prevent selection biases that could jeopardize the validity of the study results. Recent studies have shown a considerable decline in participation rates among population controls. In the INTERPHONE study, only 54% of controls participated (9); participation was further shown to be selective with respect to phone use, complicating the interpretation of study results (10, 18). Given the age range in the current study, we expected selective participation to be an issue since young people have distractions that may prevent their participation. Young adults’ participation is further complicated by ethics board requirements that require parental approval to participate (generally at ages 10–18, depending on local ethics legislation). Germany, the only center to recruit both hospital- and population-based controls, has much higher participation rates among hospital-based controls compared to population-based controls. This indicates that compliance rates are indeed much higher when using hospital-based controls (as opposed to population-based controls) and that our choice of hospital-based controls may reduce selection bias caused by low control participation rates.

Appendicitis patients were ultimately chosen as controls as this is a common disease among subject in the age range of the study, neither related to mobile phone use nor to socioeconomic status. Known risk factors for acute appendicitis include age (peak: 10–19 years), sex, ethnicity/race, family history of appendicitis, infection, seasonal variation, and having cystic fibrosis (19, 20). This limited number of risk factors, unrelated to any exposure of interest in our study, ensures that unlike other hospital-based controls, appendicitis controls are the most likely to represent the general population from which the cases arise.

## Recruitment of Cases and Controls

**Figure 1:**
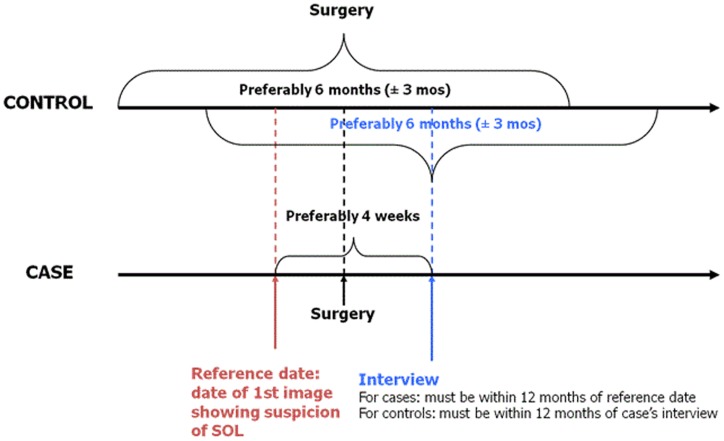
**MOBI-Kids key dates for case and control matching and eligibility**.

Because of the severity of brain tumors, rapid case ascertainment is critical. Active identification of eligible cases and controls is accomplished through contact with neurosurgery, radiology, and oncology units for cases and general surgery for controls (both adult and pediatric units). Completeness of case ascertainment is assessed by periodically reviewing cancer registries and/or hospital discharge records (where available). Cases are ascertained rapidly and every effort is made to interview them as soon after diagnosis as possible to minimize non-participation and recall bias that may occur due to deteriorating cognitive abilities among cases. Controls are identified and interviewed as soon after identifying a case as possible. For logistical reasons, however, some time may lapse between identifying and interviewing a subject. To ensure strict data quality, we permit a maximum of 12 months between a case interview and his/her reference date (date of first image showing a suspicion of a space occupying lesion) and between a case and a matched control interview (Figure 1).

As of June 2014, 2,990 eligible participants (878 cases and 2,112 controls) had been identified. Participation rates range from 78 to 83% and 60 to 69% among cases and controls, respectively (range based on best and worst case scenarios regarding pending subjects’ final decisions) (Table 2). Of the 566 cases who have been interviewed, 73% have at least one identified control and 53% have two interviewed controls (Table 3). Seventy-nine percent of controls’ interviews were performed within 6 months (26% within 1 month) of the case’s interview. Three-quarters of cases were interviewed within 6 months of their diagnosis, with 55% being interviewed within 3 months (Table 3). The study population has slightly more males than females, and more participants in the youngest age range (Table 4).

**Table 2:** **Distribution of the study population by participant status (cases and controls) as of June 2014**.

	Cases	Controls	Total
Identified as eligible	878	2112	2990
Agreed to participate	686	1275	1961
Interviewed	566	1074	1640
Refused	84	491	575
Doctor refused; dead; too ill (no proxy available)	11	0	11
Other reason for non-participation	10	13	23
Unable to locate	45	159	204
Pending confirmation	42	174	216
*Participation rates^a^*	*78–83%*	*60–69%*	*65–73%*

*^a^The ranges represent the worst and best case scenarios depending on response status; lower bound includes only current agreed, upper bound assumes all pending agree*.

**Table 3:** **Distribution of time interval between case and matched controls interviews and diagnostic date as of June 2014**.

	n (%)
**DISTRIBUTION OF INTERVAL (IN MONTHS) BETWEEN INTERVIEWS OF CASES AND INTERVIEWS OF CONTROLS**	
>6 m before case	13 (2)
6–1 m before case	63 (8)
±1 m	189 (26)
1–6 m after case	334 (45)
6–12 m after case	143 (19)
**DISTRIBUTION OF DELAYS IN MONTHS BETWEEN DIAGNOSIS AND INTERVIEW – CASES**	
<1 m	176 (31)
1–3 m	137 (24)
3–6 m	104 (19)
6–12 m	149 (26)
**STATUS OF MATCHING PAIRS AND TRIPLETS FOR CASES AGREED TO PARTICIPATE**	
Matched triplets^a^	34 (6)
Matched pairs^a^	14 (2)
No matching yet	150 (26)
Completed pairs	68 (12)
Completed triplets	300 (53)

*^a^Identified but interviews are not yet completed*.

**Table 4:** **Main characteristics of interviewed cases and controls in MOBI-Kids as of June 2014**.

	Total		Cases		Controls	
	(1640)		(566)		(1074)	
	n	%	n	%	n	%
Country						
Australia	39	2.4	14	2.5	25	2.3
Austria	9	0.5	5	0.9	4	0.4
Canada	9	0.5	5	0.9	4	0.4
France	147	9.0	63	11.1	84	7.8
Germany	152	9.3	71	12.5	81	7.5
Greece	149	9.1	42	7.4	107	10.0
India	2	0.1	2	0.4	0	0.0
Israel	187	11.4	65	11.5	122	11.4
Italy	297	18.1	106	18.7	191	17.8
Japan	198	12.1	16	2.8	182	16.9
Korea	62	3.8	23	4.1	39	3.6
New Zealand	2	0.1	2	0.4	0	0.0
Spain	360	22.0	145	25.6	215	20.0
The Netherlands	27	1.6	7	1.2	20	1.9
Sex
Males	903	55.1	307	54.2	596	55.5
Females	737	44.9	259	45.8	478	44.5
Age (years)
10–14	661	40.3	221	39.0	440	41.0
15–19	579	35.3	209	36.9	370	34.5
20–24	400	24.4	136	24.0	264	24.6
Interview type
Self-respondent	1031	62.9	312	55.1	719	66.9
Proxy respondent	16	1.0	16	2.8	NA	NA
Parent respondent	158	9.6	71	12.5	87	8.1
Both (self + parent)	424	25.9	160	28.3	264	24.6
Other	11	0.7	7	1.2	4	0.4

## Proxies

The core protocol specifies that proxies (preferably the parents) will be approached if a case has passed away or is too ill to respond to questions. Conversely, no proxies are approached for controls since in this age group the number of controls deceased or too ill to respond is expected to be minimal, therefore, not introducing any selection bias. However, if the study subject is young and/or their parents prefer to be present, the parents may help answer the questionnaire (for both cases and controls). Only 3% of cases needed a proxy interview because they were too ill or had passed away; however, an additional 40% of cases were interviewed with a parent or guardian either because they were young or the family preferred to be present (results not shown).

## Ethics Committees

Ethics approvals for conducting the study were obtained in each country, usually in each participating hospital (Table 1). As MOBI-Kids involves both adults and children, consent is given by the subject, parent/guardian, or both according to age and local ethics committee requirements. All subjects (and/or parents/guardians) are asked to sign an informed consent form before participating in the study.

In recent years, ethics approvals have become more complex. Seven centers had to obtain ethics approvals from each individual hospital (median number of ethics approvals per country: 16; range: 1 national ethics committee in France to 69 individual approvals in Spain) (Table 1). In Austria, ethics requirements changed during the study period, requiring study staff to stop recruiting participants and to submit applications at county-level ethics committees, resulting in a loss of over a year of fieldwork. Ethics requirements have become more restrictive. Several centers are not allowed to recruit or even contact patients until they have signed the informed consent form, placing the responsibility for recruitment on already overworked doctors/hospital staff. Besides making logistical aspects of MOBI-Kids more difficult, burdensome ethics requirements could have significant implications on other epidemiological studies as it means the denominator is uncertain – we have to rely on busy clinicians to carefully record all eligible cases who were recruited to join the study and to follow-up on their recruitment. Furthermore, recruitment of controls by doctors or hospital staff is especially onerous as appendicitis patients are only in the hospital for a short time, and further contact with them is limited. It is impractical to expect busy hospital staff to recruit controls following a rigorous epidemiological study protocol, but, given the strict ethics requirements, several countries are left with no choice in this regard. As mentioned above, these issues contributed to an appreciable reduction in the number of cases recruited relative to the originally projected number.

## Questionnaires and Study Instruments

Trained interviewers administer either an electronic or paper version of a detailed questionnaire developed from INTERPHONE and other recent brain tumor and/or mobile phone studies (21–24), modified to include technological advancements, simplified for younger subjects, and optimized based on pilot testing in several countries. The main questionnaire includes demographic variables; use of communication technologies (mobile phones, cordless telephones, and Wi-Fi); exposure to non-communication sources of ELF and RF-EMF; occupational history including occupational exposures to EMF; and other possible risk factors for brain tumors (e.g., medical history and radiation exposures).

The detailed section on mobile phone use is administered only to subjects answering “yes” to the screening question about ever having been a “regular mobile phone user” – defined as making on average at least one call per week for 3 months or more. Questions are asked about initial and current use, including number and duration of voice calls; use of hands-free kits, speaker phone, and/or Bluetooth headsets; laterality of use; proportion of time using phone in urban/suburban/rural areas; and other phone usage [i.e., number of SMS and other messaging apps, and time spent using email, Internet, and voice over Internet Protocol (VoIP) (e.g., Skype)]. Subjects are also asked about changes in phone use to further characterize their phone use history. All makes and models of phones used are identified with the assistance of a custom-made searchable database containing over 6,500 phones.

Preliminary analyses show that <2% of the questions in the main questionnaire have 5% or greater “do not know” responses, indicating that the questionnaire is generally clear to the entire study population (results not shown).

In addition to the subject’s questionnaire, parents (preferably the mother) are asked about maternal smoking history and other exposures before conception, during the pregnancy and the first trimester of life of the child, as well as about the pregnancy itself, the child’s delivery, and her/his school history. Parental occupational histories are collected for both parents. Clinical data regarding the disease status, surgery, pathology, imaging needed for diagnosis verification, and tumor classification are collected from all available medical files.

## Non-Respondents

In case of refusals, where access to study subjects is allowed by ethics committees, subjects are asked to complete a short non-response questionnaire about mobile phone use and maternal education level. This questionnaire will be used to evaluate possible selection bias among participants.

## Validation Studies

Validation of self-reported phone use is conducted by comparing responses of consenting subjects to network operator records. In addition, Mobi-Expo, a separate but complementary study of volunteers as well as a group of MOBI-Kids controls using software-modified smartphones (SMPS), collects self-reported phone use as well as use patterns, including laterality and data use recorded by the SMPS. Mobi-Expo will provide important information about mobile phone usage patterns in young people recorded by the SMSP as well as a means of validating self-reported mobile phone use.

## Tumor Localization

Neuroradiologists will locate each case’s tumor on a generic 3D head model using cases’ MRI or CT scans, similar to what was done in INTERPHONE (25). Unlike INTERPHONE, however, there are four head models corresponding to three child-sized and one adult-sized head (according to age) and much more sophisticated exposure estimation.

## Exposure Assessment

Collecting reliable valid data on complex and rapidly changing patterns of exposures, while minimizing recall bias and errors, has been a significant challenge in MOBI-Kids. Therefore, considerable effort was invested in developing the questionnaire’s exposure sections based on an extensive ELF and RF-EMF measurement and modeling campaign (26) and experience gained from INTERPHONE and expert opinions. Much thought was given to minimizing recall biases (e.g., use of prompts and a searchable mobile phone database to facilitate identification of phones used; first asking questions about present use and then previous use). In addition, we allow a maximum of 12 months between the case and control interviews (and also between the case’s reference date and interview) to minimize differential recall bias between cases and controls.

MOBI-Kids includes numerous validation checks to ensure the accuracy of the questionnaire responses. The development of an electronic mobile phone database containing details on several thousand mobile phones has resulted in a substantial reduction in unknown phone models. Before launching the electronic mobile phone database in June 2011, 36% of phones were unknown (that is, subjects could not identify the make and/or model), whereas only 16% of phones since June 2011 are unknown, clearly demonstrating the benefit of a searchable mobile phone database to assist in identifying mobile phones, a factor that is an important in determining the exposure from the phones.

Due to the rapid increase in the use of intermediate frequency (IF) technologies, some questions about sources of IF were added to the questionnaire in early 2013; the job histories will also be coded for IF-exposed jobs. This capability to address emerging issues in non-ionizing radiation highlights the flexibility of exposure assessment in the case–control approach, to address changes in types of exposures in a rapidly evolving technological field.

## Study Power

As discussed above, despite our best efforts to reach the original expected sample size of approximately 2,000 cases, the revised projected number of case is just under 1,000. However, preliminary results on mobile phone use among controls indicate that 77 and 83% of males and females, respectively, were defined as ever using a mobile phone regularly (data not shown). In keeping with the INTERPHONE study, subjects who had used a mobile phone for <1 year were considered “never” regular users. Further, approximately 14% of all subjects in MOBI-Kids have used a mobile phone for 10 years or longer, the threshold for long-term use in INTERPHONE. As this was a higher proportion than originally expected, our power calculations were revised based on the updated expected number of subjects and updated exposure indicators. Assuming that 971 cases are included in matched analyses, the study has 79% power to detect an increased risk of 40% [the estimated increase in the risk of glioma seen in the highest decile of phone use in INTERPHONE (10)], assuming 10% have used a mobile phone for 10 years or longer; power increases to 90% assuming 15% are “long-term” mobile phone users.

## Statistical Analysis

The main analysis will be based on standard statistical methods for analysis of case-control studies (18). As in INTERPHONE, this study will use two approaches to characterize exposure from mobile phones. The first will be based on self-reported history of use (including number of years of use, duration of calls, laterality, and other exposure metrics) calibrated against objective data, while the second will expand on INTERPHONE’s methods to estimate the amount of RF energy absorbed in the brain at the tumor location (25, 27) (as well as brain exposure to IF and ELF when possible).

## Conclusion

In spite of its challenges, the advantages of MOBI-Kids include its large sample size – it will be the largest study to date on this topic in young people – covering 14 participating countries. Subjects are being identified and recruited in a time period in which mobile phone use in young people has become more prevalent, thus, increasing the statistical power and overall representativeness and generalizability of the results. In addition, MOBI-Kids includes extensive exposure assessment work and validation studies using both historical provider records and SMPS to counteract potential recall bias. Despite the various challenges faced by the study team (which have implications for other epidemiological studies), our experience thus far in developing and implementing the study protocol indicates that MOBI-Kids is feasible and will generate results contributing to the understanding of potential brain tumor risks associated with use of mobile phones and other wireless communication technologies among young people.

## MOBI-Kids Consortium

Centre for Research in Environmental Epidemiology (CREAL): Elisabeth Cardis, Chelsea Eastman Langer, Gema Carretero, L. Kincl (now at: College of Public Health and Human Sciences, Oregon State University). Martine Vrijheid, Alex Albert, Laura Argenté, and Patricia de Llobet.

Australia: Monash University – Malcolm R. Sim, Rosa Schattner, Geza Benke; Telecom Institute for Child Health Research Western Australia – Elizabeth Milne; University of Sydney – Bruce Armstrong.

Austria: Medical University of Vienna – Michael Kundi, Hans-Peter Hutter, and Adelheid Woehrer.

Canada: Department of Epidemiology and Community Medicine, University of Ottawa – Daniel Krewski, Charmaine Mohipp, Franco Momoli, and D. Bedard; British Columbia Cancer Agency – John Spinelli, M. Elwood, and A. Lai; Cancer Care Ontario – Paul Ritvo, L. Stefanyk and Tina Changoor.

France: French National Registry of Childhood Solid Tumors, CHU, Nancy – Brigitte Lacour, Thomas Remen, and Dominique Delmas; IFSTTAR – Martine Hours; CHU Montpellier – Luc Bauchet; Orange – Joe Wiart, E. Conil, N. Varsier, T. Sarrebourse, and Abdelhamid Hadjem.

Germany: Institute and Outpatient Clinic for Occupational, Social and Environmental Medicine, University Hospital of Munich, Ludwig Maximilian University of Munich – Katja Radon, Tobias Weinmann, Sabine Heinrich, Swaantje Klostermann, and Vanessa Kiessling.

Greece: National and Kapodistrian University of Athens – Eleni Petridou, Evdoxia Bouka, and Paraskevi Panagopoulou.

India: Tata Memorial Hospital – Rajesh Dikshit and Rajini Nagrani.

Israel: Cancer and Radiation Epidemiology Unit, Gertner Institute – Siegal Sadetzki, Revital Bruchim, Angela Chetrit, G. Hirsh-Yechezkel A. Zultan, K. Manor, T. Ben-Tal Grinshpan, L. Aslanov, and Hadas Even-Nir.

Italy: Unit of Cancer Epidemiology, Department of Medical Sciences, University of Turin – Franco Merletti, Milena Maule, and Enrica Migliore; Neuroepidemiology Research Unit, Fondazione I.R.C.C.S Istituto Neurologico Carlo Besta, Milan – Graziella Filippini and M. Farinotti; Institute for the Study and Prevention of Cancer, Unit of Occupational and Environmental Epidemiology, Florence – Lucia Miligi; Occupational Health Unit, Sant’Orsola-Malpighi Polyclinic and University of Bologna – Stefano Mattioli.

Japan: Tokyo Women’s Medical University – Naohito Yamaguchi, Noriko Kojimahara, D. Furushima, K. Kiyohara, N. Tattybek, M. Shimoyamada, and S. Sato; Tokyo Metropolitan University – Masao Taki and K. Wake.

Korea: Dankook University College of Medicine – Mina Ha, Kyung-Hwa Choi, and Y.J. Lee; Electronics and Telecommunications Research Institute – Ae-Kyoung Lee and H.-D. Choi.

New Zealand: Massey University – Andrea ’t Mannetje and Amanda Eng; University of Auckland – Alistair Woodward.

Spain: University of Huelva, Andalucía – Juan Alguacil and A. Zumel; Carlos III Institute of Health – N. Aragonés, M. Pollán, B. Pérez-Gómez, E. Ferreras, and A. Sierra; University of Valencia – Maria Morales Suárez-Varela, I. Gavidia, and Agustín Llopis-González.

The Netherlands: Institute for Risk Assessment Sciences, Division Environmental Epidemiology, Utrecht University – Hans Kromhout, Roel Vermeulen, and Geertje Goedhart.

United Kingdom: Physical Dosimetry Department, Public Health England – Myron Maslanyj, S. Mann, C. Calderon, D. Addison, T. Mee, and R. Findlay (now at: EMFcomp, Wantage, Oxfordshire, UK).

## Conflict of Interest Statement

Daniel Krewski has conducted contract work for the federal government of Canada (specifically, the Public Health Agency of Canada and Industry Canada) involving systematic review and summary of scientific information on potential health effects of radiofrequency fields. Malcolm R. Sim – wife had shares of Cell Phone Company. Masao Taki’s department received a grant to support numerical modeling work under a university–industry partnership. Joe Wiart works at Whist Laboratory funded by Orange. None of this funding was used to support the research described in this paper. The other authors declare that they have no conflict of interest.

## Supplementary Material

The Supplementary Material for this article can be found online at http://www.frontiersin.org/Journal/10.3389/fpubh.2014.00124/abstract
