# Supplementary material for: The MOBI-Kids Study Protocol: Challenges in Assessing Childhood and Adolescent Exposure to Electromagnetic Fields from Wireless Telecommunication Technologies and Possible Association with Brain Tumor Risk
Source: Front Public Health. 2014 Sep 23;2:124. doi: 10.3389/fpubh.2014.00124 (PMC4172002; doi:10.3389/fpubh.2014.00124)
Supplement: Supplementary file 1 [file DataSheet_1.PDF]

Appendix A:

List of eligible brain tumour topologies and morphologies

Topography and Behavior

	Topography codes					Morphology codes			
	ICD-9		ICD-10		ICD-O3	ICD-O 3rd edition			
						ICD-O / 0 / 1		ICD-O / 3	
**Glioma**	191.0[1]	Cerebrum, except lobes and ventricles	C71.0[1]	Cerebrum, except lobes and ventricles	C71.0	9383/1	Subependymoma	9380/3	Glioma, NOS
						9384/1	Subependymal giant cell astrocytoma	9381/3	Gliomatosis cerebri
	191.1	Frontal lobe	C71.1	Frontal lobe	C71.1			9382/3	Oligoastrocytoma
	191.2	Temporal lobe	C71.2	Temporal lobe	C71.2	9394/1	Myxopapillary ependymoma	9382/3	Oligoastrocytoma, anaplastic
	191.3	Parietal lobe	C71.3	Parietal lobe	C71.3				
	191.4	Occipital lobe	C71.4	Occipital lobe	C71.4	9421/1	Pilocytic astrocytoma	9382/3	Mixed glioma
	191.5	Ventricles	C71.5	Cerebral ventricle	C71.5	9442/1	Gliofibroma	9391/3	Ependymoma
	191.6	Cerebellum	C71.6	Cerebellum	C71.6			9392/3	Ependymoma, anaplastic
	191.8	Other parts of brain	C71.7[5]	Brain stem	C71.7			9393/3	Ependymoma
			C71.8	Overlapping lesion of brain	C71.8			9400/3	Diffuse astrocytoma
	191.9	Brain, unspecified	C71.9	Brain, unspecified	C71.9			9401/3	Astrocytoma, anaplastic
	192.9[2]	Nervous system, part unspecified[3]	C72.8[2]	Overlapping lesion	C72.8			9410/3	Diffuse astrocytoma
			C72.9	Central nervous system, unspecified	C72.9			9411/3	Diffuse astrocytoma
	237.5[4]	Brain and spinal cord						9420/3	Diffuse astrocytoma
			D43.0[4]	Brain, supratentorial				9424/3	Pleomorphic xanthoastrocytoma
			D43.1[4]	Brain, infratentorial					
			D43.2[4]	Brain, unspecified				9425/3	Pilomyxoid astrocytoma
			D43.7[4]	Other parts of central nervous system				9440/3	Glioblastoma
								9441/3	Giant cell glioblastoma
								9442/3	Gliosarcoma
								9450/3	Oligodendroglioma
								9451/3	Oligodendroglioma, anaplastic
								9460/3	Oligodendroblastoma

[1] ICD-9 191/ICD-10 C71: Malignant neoplasm of brain

[2] ICD-9 192: Malignant neoplasm of other and unspecified parts of nervous system; ICD-10 C72: Malignant neoplasm of spinal cord, cranial nerves, and other parts of central nervous system

[3] ICD-9 192.9: Should not occur, but is sometimes coded if the tumour spreads over larger areas (coded as C71.8 in ICD-10)

[4] ICD-9 237.5/ICD-10 D43.x: Should not occur with glioma (refers to brain tumours of uncertain behaviour)

[5] This topography only for brain stem glioma (pontine glioma)

Topography and Behavior

	Topography codes					Morphology codes			
	ICD-9		ICD-10		ICD-O3	ICD-O 3rd edition			
						ICD-O / 0 / 1		ICD-O / 3	
**Choroid plexus tumours**	191.5	Ventricles	C71.5	Ventricles	C71.5	9390/0	Choroid plexus papilloma	9390/3	Choroid plexus carcinoma
	225.0	Brain (benign neoplasm)	D33.0[6]	Brain, supratentorial					
			D33.1	Brain, infratentorial		9390/1	Atypical choroid plexus papilloma		
	237.5	Brain (neoplasm of uncertain behaviour)	D43.0[7]	Brain, supratentorial					
			D43.1	Brain, infratentorial					
**Other neuroepithelial tumours**	191.0	Cerebrum, except lobes and ventricles	C71.0	Cerebrum, except lobes and ventricles	C71.0	9431/1	Angiocentric glioma	9430/3	Astroblastoma
						9444/1	Chordoid glioma of the third ventricle		
	191.1	Frontal lobe	C71.1	Frontal lobe	C71.1				
	191.2	Temporal lobe	C71.2	Temporal lobe	C71.2				
	191.3	Parietal lobe	C71.3	Parietal lobe	C71.3				
	191.4	Occipital lobe	C71.4	Occipital lobe	C71.4				
	191.5	Ventricles	C71.5	Cerebral ventricle	C71.5				
	191.6	Cerebellum	C71.6	Cerebellum	C71.6				
	191.8	Other parts of brain	C71.8	Overlapping lesion of brain	C71.8				
	191.9	Brain, unspecified	C71.9	Brain, unspecified	C71.9				
	192.9	Nervous system, part unspecified	C72.8	Overlapping lesion					
	237.5	Brain and spinal cord	C72.9	Central nervous system, unspecified					
			D43.0	Brain, supratentorial					
			D43.1	Brain, infratentorial					
			D43.2	Brain, unspecified					
			D43.7	Other parts of central nervous system					

[6] ICD-10 D33: Benign neoplasm of brain and other parts of central nervous system

[7] ICD-10 D43: Neoplasm of uncertain or unknown behaviour of brain and central nervous system

Topography and Behavior

	Topography codes					Morphology codes			
	ICD-9		ICD-10		ICD-O3	ICD-O 3rd edition			
						ICD-O / 0 / 1		ICD-O / 3	
**Neuronal and mixed neuronal-glial tumours**	191.0	Cerebrum, except lobes and ventricles	C71.0	Cerebrum, except lobes and ventricles	C71.0	8680/0	Paraganglioma[9], benign	8680/3	Paraganglioma[9], malignant
								9505/3	Anaplastic ganglioglioma
	191.1	Frontal lobe	C71.1	Frontal lobe	C71.1	8680/1	Paraganglioma[9], NOS		
	191.2	Temporal lobe	C71.2	Temporal lobe	C71.2	8690/1	Jugular paraganglioma		
	191.3	Parietal lobe	C71.3	Parietal lobe	C71.3	9412/1	Desmoplastic infantile astrocytoma/ ganglioglioma		
	191.4	Occipital lobe	C71.4	Occipital lobe	C71.4				
	191.5	Ventricles	C71.5	Cerebral ventricle	C71.5				
	191.6	Cerebellum	C71.6	Cerebellum	C71.6	9413/0	Dysembryoplastic neuroepithelial tumour		
	191.8	Other parts of brain	C71.8	Overlapping lesion of brain	C71.8				
						9492/0	Gangliocytoma		
	191.9	Brain, unspecified	C71.9	Brain, unspecified	C71.9	9493/0	Dysplastic gangliocytoma of cerebellum (Lhermitte-Duclos)		
	192.9	Nervous system, part unspecified	C72.8	Overlapping lesion	C72.8				
			C72.9	Central nervous system, unspecified	C72.9				
	225.0[8]	Brain							
	225.8	Other specified sites of nervous system			C75.5	9505/1	Ganglioglioma		
			D33.0	Brain, supratentorial		9506/1	Central neurocytoma		
	225.9	Nervous system, part unspecified	D33.1	Brain, infratentorial		9509/1	Papillary glioneuronal tumour		
			D33.2	Brain, unspecified					
	237.5	Brain and spinal cord	D43.0	Brain, supratentorial		9509/1	Rosette-forming glioneuronal tumour of the fourth ventricle		
			D43.1	Brain, infratentorial					
			D43.2	Brain, unspecified					
			D43.7	Other parts of central nervous system					

[8] ICD-9 225: Benign neoplasm of brain and other parts of nervous system

[9] Only from glomus tympanicum and glomus jugulare

Topography and Behavior

	Topography codes					Morphology codes			
	ICD-9		ICD-10		ICD-O3	ICD-O 3rd edition			
						ICD-O / 0 / 1		ICD-O / 3	
**Embryonal tumours**	191.0	Cerebrum, except	C71.0	Cerebrum, except	C71.0			9470/3	Medulloblastoma
		lobes and ventricles		lobes and ventricles				9471/3	Desmoplastic/nodular
	191.1	Frontal lobe	C71.1	Frontal lobe	C71.1				medulloblastoma
	191.2	Temporal lobe	C71.2	Temporal lobe	C71.2			9472/3	Medullomyoblastoma
	191.3	Parietal lobe	C71.3	Parietal lobe	C71.3			9473/3	CNS primitive
	191.4	Occipital lobe	C71.4	Occipital lobe	C71.4				neuroectodermal tumour
	191.5	Ventricles	C71.5	Cerebral ventricle	C71.5			9474/3	Anaplastic medulloblastoma
	191.6	Cerebellum	C71.6	Cerebellum	C71.6				
	191.8	Other parts of brain	C71.8	Overlapping lesion	C71.8			9480/3	Cerebellar sarcoma, NOS
				of brain				9490/3	CNS primitive
	191.9	Brain, unspecified	C71.9	Brain, unspecified	C71.9				neuroectodermal tumour
	192.9	Nervous system, part	C72.8	Overlapping lesion	C72.8			9500/3	CNS primitive
		unspecified	C72.9	Central nervous	C72.9				neuroectodermal tumour
				system, unspecified				9501/3	CNS primitive
									neuroectodermal tumour
**Tumours of cranial and**	192.0	Cranial nerves	C72.2	Olfactory nerve	C72.2	9540/0,1	Neurofibroma	9540/3	Malignant peripheral nerve
**paraspinal nerves**	225.1	Cranial nerves	C72.3	Optic nerve	C72.3	9550/0	Neurofibroma		sheath tumour (epithelioid,
			C72.4	Acoustic nerve	C72.4	9560/0,1	Schwannoma (cellular,		mesenchymal, glandular)
			C72.5	Other and	C72.5		plexiform, melanotic)	9571/3	Malignant perineurinoma
				unspecified cranial		9571/0	Perineurioma		
				nerves					
			D33.3	Cranial nerves					

Topography and Behavior

	Topography codes					Morphology codes			
	ICD-9		ICD-10		ICD-O3	ICD-O 3rd edition			
						ICD-O / 0 / 1		ICD-O / 3	
**Tumours of the meninges**	192.1<br>225.2<br>237.6	Cerebral meninges<br>Cerebral meninges<br>Meninges	C70.0<br><br>D32.0<br>D42.0	Cerebral meninges<br><br>Cerebral meninges<br>Cerebral meninges	C70.0<br>C70.9	9530/0,1<br>9531/0<br>9532/0<br>9533/0<br>9534/0<br>9535/0<br>9537/0<br>9538/1<br><br>9539/1	Meningioma<br>Meningioma<br>Meningioma<br>Meningioma<br>Meningioma<br>Meningioma<br>Meningioma<br>Atypical, chordoid, clear cell meningioma<br>Atypical, chordoid, clear cell meningioma	9530/3<br><br>9538/3<br><br>9539/3	Papillary, rhabdoid, anaplastic meningioma<br>Papillary, rhabdoid, anaplastic meningioma<br>Meningeal sarcomatosis
**Other neoplasms**						9161/1	Haemangioblastoma[10]		

[10] Eligible if not associated with Von Hippel-Lindau syndrome and located in the CNS
